# Nursing care for a patient with intracerebral hemorrhage complicated with toxic epidermal necrolysis: a case report

**DOI:** 10.3389/fmed.2025.1587979

**Published:** 2025-07-18

**Authors:** Doudou Zhang, Qiuping Zhang, Hanhua Li, Wen Lai, Pingyun Chen

**Affiliations:** Department of Burn and Wound Repair Surgery, Guangdong Provincial People’s Hospital (Guangdong Academy of Medical Sciences), Southern Medical University, Guangzhou, Guangdong, China

**Keywords:** intracerebral hemorrhage, toxic epidermal necrolysis, infection, nursing, lumbar cistern drainage

## Abstract

This study summarizes the nursing care for a patient with intracerebral hemorrhage complicated with toxic epidermal necrolysis, focusing on infection prevention and control. Advanced health assessment showed that the primary nursing issue was a high risk of infection. Nursing interventions were implemented to prevent and control infection in various systems, emphasizing four key aspects: respiratory management after tracheostomy, lumbar cistern drainage care, skin management, and nutritional support. The patient’s skin healed, infection indicators decreased, and no secondary infections occurred. On the 24*^th^* day after admission, the patient was transferred to the neurosurgery department for ventriculoperitoneal shunt surgery. The patient was transferred to a rehabilitation hospital 21 days after surgery.

## 1 Introduction

Hypertensive intracerebral hemorrhage is one of the most common critical diseases, with severe cases often leading to coma. Among elderly patients, pulmonary function decline combined with prolonged bed rest significantly increases the risk of infection. The extensive use of antibiotics to treat these infections frequently leads to adverse drug reactions due to the concurrent administration of multiple antimicrobial agents (1), further compromising immune function. Stevens-Johnson syndrome (SJS) is a predominantly drug-induced, life-threatening severe cutaneous adverse reaction (2). Toxic epidermal necrolysis (TEN), the most severe form of SJS, is primarily triggered by medications such as antibiotics, antipyretics/analgesics, sulfonamides, and barbiturates. Clinically, it is characterized by widespread keratinocyte death, presenting with blistering, mucosal sloughing, and epidermal necrosis. The fulminant course of TEN involves extensive skin exfoliation, resulting in substantial protein loss and fluid-electrolyte imbalance. Severe cases may lead to death from secondary infections, with reported mortality rates ranging from 14.8% to 48.0% (3). In patients with TEN, the loss of the skin’s barrier function confers a significant predisposition to secondary infections (4). Sepsis arising from these infections is the leading cause of mortality in severe cases, with mortality rates approaching 50% in the elderly population (5). Therefore, infection prevention and control are critical to improving patients’ outcomes. In October 2024, our department admitted an elderly patient with intracerebral hemorrhage complicated by severe TEN. Through advanced health assessment, a high risk of infection was identified as the primary nursing issue. Based on infection prevention and control across various systems, nursing interventions focused on four key areas: respiratory management after tracheostomy, lumbar cistern drainage care, skin management, and nutritional support. The outcomes were remarkable, the patient’s skin healed, indicators of infection were markedly decreased, and no sign of secondary infection was observed. On the 24th day after admission, the patient was transferred to the neurosurgery department for ventriculoperitoneal shunt placement. Twenty-one days postoperatively, the patient was discharged to a rehabilitation hospital. The detailed nursing process is reported as follows:

## 2 Case description

### 2.1 General information

A 69 years-old bedbound male acutely developed an altered level of consciousness on 3 September 2024. Cranial CT at an external hospital revealed intracerebral hemorrhage (ICH) with intraventricular extension (IVH). Emergency bilateral external ventricular drain (EVD) placement and intracranial pressure (ICP) monitor insertion were performed. On the afternoon of October 7, multiple erythematous patches emerged on his trunk, managed empirically as allergic dermatitis with dexamethasone and calcium gluconate. That evening, recurrent multifocal ICH with IVH occurred, requiring resuscitation before transfer back to the EICU. From October 9, the cutaneous rash progressed, involving the chest and abdominal regions. He received intravenous dexamethasone 10 mg daily, methylprednisolone 40 mg daily, and a single dose of intravenous immunoglobulin (IVIG) 20 g. While the right abdominal rash improved, lesions on the neck, chest, and back persisted, with epidermal detachment and Nikolsky sign-positive skin sloughing, particularly on the posterior neck and back. He was transferred to our institution on October 18 for specialized management of suspected TEN, a complication arising >1 month post-ICH. Background: Non-contributory birth/origin history. No significant toxicant or occupational dust exposure. Regular lifestyle. No drug allergy history. Negative family history of diabetes, hypertension, or genetic disorders. The family demonstrates full adherence to care protocols. On admission, his vital signs were as follows: temperature 36.4°C, pulse 100 beats/min, respiration 20 breaths/min, and blood pressure 130/67 mmHg (1 mmHg = 0.133 kPa). Physical examination revealed shallow coma, bilaterally equal and round pupils with a diameter of 1 mm, sluggish light reflexes, and a Glasgow Coma Scale (GCS) score of E1V1M4. More than 70% of the patient’s skin exhibited epidermal detachment, most prominently on the head, face, neck, trunk, and upper limbs. There was minimal exudation with no extensive peeling; oral and nasal mucosa were also involved. The lumbar cistern drainage tube demonstrated poor patency, with a low output of daily cerebrospinal fluid. The patient presented with pulmonary infection, characterized by abundant thick sputum. Laboratory tests showed that white blood cell count was 24.70 × 10^9^/L, hemoglobin level was 94 g/L, platelet count was 329 × 10^9^/L, neutrophil ratio was 0.906, partial pressure of oxygen (PaO_2_) was 83.8 mmHg, partial pressure of carbon dioxide (PaCO_2_) was 41.2 mmHg, pH was 7.445, (1→3)-β-D-glucan level was 883.3 pg/mL, albumin level was 26.6 g/L, D-dimer level was 1,450 ng/mL, and interleukin-6 (IL-6) level was 33.9 pg/mL. Based on these findings, the patient was diagnosed with TEN, hydrocephalus, and pulmonary infection.

### 2.2 Treatment process and outcomes

Table 1 details the comprehensive therapeutic course from hospital admission through disease stabilization and skin lesion healing until transfer to a rehabilitation hospital at 21 days post-neurosurgical intervention.

**TABLE 1:** Treatment process and outcomes.

Time	Event
Admission day	1. Admitted to a laminar flow ward, placed on a suspension bed, and received intensive care. A critical condition notice was issued. 2. Consultations requested with neurosurgeons, clinical nutritionists, and dermatologists.
Simultaneously	3. Received intravenous methylprednisolone to treat allergy, human immunoglobulin to support the immune system, and 20% human albumin supplementation. 4. Treated with polymyxin B and fluconazole to control infection. 5. Provided enteral nutrition emulsion (short peptide type) for nutritional support.
Day 3 after admission	The patient’s condition was critical, with abundant thick sputum and a high risk of vomiting due to intracerebral hemorrhage. 1. Nasointestinal tube was placed. 2. Tracheostomy was conducted after consultation with the otolaryngology department, and the tracheostomy tube was connected to a high-flow, heated, humidified respiratory support device.
Day 5 after admission	Head CT revealed significant intracranial fluid accumulation; and lumbar cistern drainage surgery was conducted after replacing the drainage tube.
Day 7 after admission	Widespread epidermal detachment involving skin proximal to the knees, with prominent involvement of the head, face, neck, trunk, and upper extremities. Numerous blisters of varying sizes formed on the upper limbs and upper chest, with no large-scale peeling, the affected area exceeds 70% of estimated total body surface area (TBSA) (Figure 1).
Day 9 after admission	1. Cerebrospinal fluid contained flocculent material, and physical examination revealed neck stiffness. Metagenomic testing of cerebrospinal fluid showed *Staphylococcus epidermidis* infection, and blood culture revealed *Klebsiella pneumoniae* infection. In addition, isolation was implemented, the management of lumbar cistern drainage tube was reinforced, and pulmonary inflammation improved after bronchoscopy-guided sputum suction. 2. Consultation with the neurosurgery department was conducted, and antibiotic choices were switched to meropenem and linezolid.
Day 19 after admission	Most lesions on skin proximal to the knees were dried, affecting 69% of total body surface area (TBSA) as measured by Lund-Browder chart. Re-epithelialization islands are observed in 65% of TBSA, with partial overlap in affected zones (Figure 2).
On day 24 after admission	(1→3)-β-D-glucan level: 49.1 pg/mL (94.4% reduction from peak of 880 pg/mL). Serum IL-6: 5.7 pg/mL (within normal limits; reference range <7.0 pg/mL). Concomitant resolution of fever and C-reactive protein normalization support presumptive clinical cure of invasive fungal infection. Systemic inflammatory response has significantly resolved, transitioning to the tissue remodeling phase of infection-related damage, cerebrospinal fluid culture was negative, the skin had healed, and consciousness recovered. The patient was transferred to the neurosurgery department for surgical treatment.
Postoperative day 21	Transferred to a rehabilitation hospital.

**FIGURE 1:**
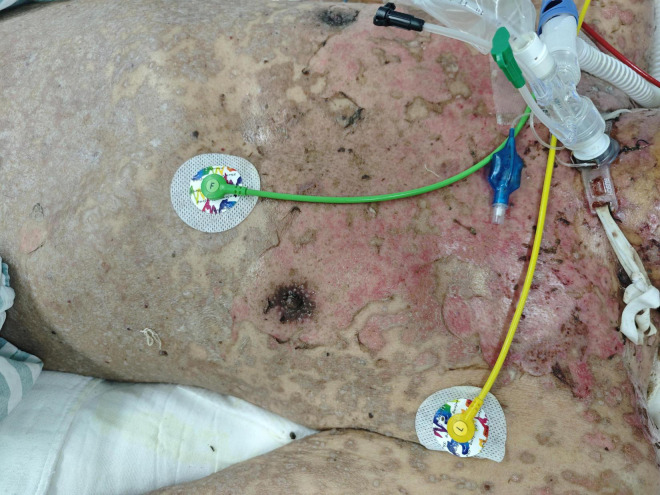
Skin lesions of the patient before treatment.

**FIGURE 2:**
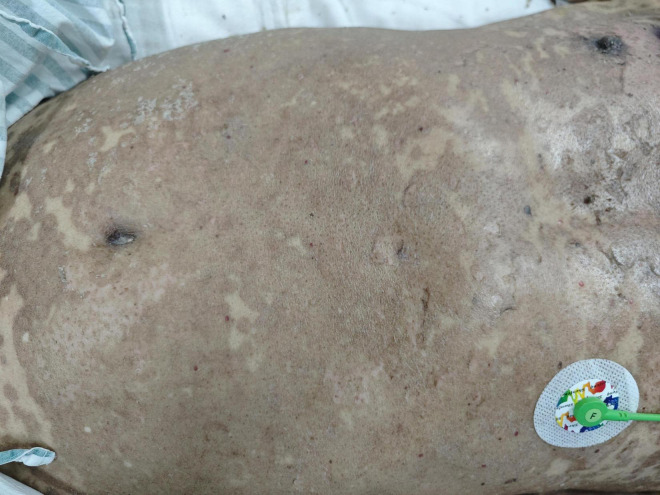
Skin lesions of the patient after treatment.

## 3 Nursing care

### 3.1 Prevention and control of respiratory infections

The patient’s condition was critical, with abundant and thick sputum, a history of intracerebral hemorrhage, a prolonged disease course, and a high risk of vomiting. The patient underwent tracheostomy after admission. Postoperatively, the airway was directly exposed to the external environment, compromising its integrity and continuity, which significantly increased the risk of lower respiratory tract infections, mortality, and disability (6). Studies have shown (7) that in patients with severe disease and impaired immune response, the incidence of pulmonary infection following tracheostomy can be as high as 43.6%. Therefore, effective management of the airway is particularly critical after tracheostomy. The key nursing measures are as follows (Table 2).

**TABLE 2:** Key points in the care of respiratory infections.

Nursing measures	Specific content
High-flow oxygen therapy	The tracheostomy tube was connected to a high-flow oxygen therapy device. 1. Parameter settings: Temperature was set at 37°C, oxygen flow was set at 40–50 L/min, and oxygen concentration was adjusted based on blood oxygen saturation to maintain SpO_2_ ≥ 95%. 2. Effect: Fully humidifies and warms inhaled air (16), increasing sputum discharge and reducing pulmonary moist rales.
Sputum suction	Method: 1. Closed suction method combined with fiberoptic bronchoscopy-guided suction. 2. Regular stepwise and segmented suction every 2–3 h, based on airway sputum volume and mucosal detachment, covering “bilateral nasal cavities-oral cavity-tracheal tube-airway.” Effect: 1. Prevents contamination and cross-infection via tracheostomy tube, thus airborne pathogens cannot enter the lungs. 2. Decreased risk of pulmonary infection (17, 18).
Nutritional support	1. Method: Nasointestinal tube placement for post-pyloric nutritional support. 2. Effect: Reduced incidence of reflux, aspiration, and aspiration pneumonia (19).
Anti-infective therapy	The choice of antibiotics was adjusted based on drug sensitivity results, switching from polymyxin B and fluconazole to meropenem and linezolid.
Tracheostomy tube site care	Dressing changes were conducted based on skin condition, using different dressings at different stages to prevent exogenous infections. 1. Skin damage phase: a vaseline gauze was used as a barrier, and recombinant human acidic fibroblast growth factor spray was applied to accelerate the healing process around the neck. 2. Exudation phase: Foam dressings were applied, exudates were monitored, and dressings were replaced when necessary. 3. Scabbing phase: a sterile gauze was used to keep the area dry. Physiological saline was applied to moisten the area before gently removing the gauze when scabs adhered to the gauze.
Assessment of surgical feasibility	Neurosurgical intervention was deemed feasible based on the following: 1. Normal body temperature with no fever. 2. Decreased pulmonary moist rales and absence of aspiration, reflux, or aspiration pneumonia. 3. Significant decrease in infection indicators: (1→3)-β-D-glucan level equal to 49.1 pg/mL and IL-6 level equal to 5.7 pg/mL. Concomitant resolution of fever and C-reactive protein normalization support presumptive clinical cure of invasive fungal infection. Systemic inflammatory response has significantly resolved, transitioning to the tissue remodeling phase of infection-related damage.

### 3.2 Prevention and control of intracranial infection

The primary nurse observed white flocculent material in the lumbar cistern drainage fluid on the second day after admission and promptly reported it to the physician. Cerebrospinal fluid was collected for metagenomic testing, which indicated *Staphylococcus epidermidis* infection. Intracranial infection was suspected after consultation with the attending physician and neurosurgeons. The lumbar cistern drainage puncture tube was replaced, and continuous drainage was maintained, which effectively treated intracranial infection (8). The key nursing measures were as follows (Table 3).

**TABLE 3:** Key points in the care of intracranial infection.

Nursing measures	Specific content
Routine care	The patient’s pupils, consciousness, and vital signs were closely monitored.
Anti-infective treatment	Antibiotics were adjusted based on the results of drug sensitivity, switching to meropenem and linezolid. Combined with continuous lumbar cistern drainage, this approach improved clinical outcomes (20).
Strict flow control	The lumbar cistern drainage volume was maintained at 200–300 mL/day with a uniform flow rate. This strategy helped normalize inflammatory cytokine levels in cerebrospinal fluid and improve the patient’s prognosis (21).
Strict aseptic technique	1. The puncture site was kept sterile, and disinfected twice daily with povidone-iodine solution for 30 s each time. The puncture site was covered with sterile transparent dressings. Sterile thin foam dressings were used over the puncture site during the exudation phase. 2. When daily recording of lumbar cistern drainage volume, the connection between the drainage tube and drainage bag was disinfected with povidone-iodine. Thereafter, cerebrospinal fluid samples were collected and analyzed (22).
Reducing catheter-associated infections	During suctioning, repositioning, and dressing changes, the drainage tube was promptly clamped to prevent the backflow of cerebrospinal fluid (23).
Assessment of surgical feasibility	On the 24^th^ day after admission, the patient was transferred to the neurosurgery department for surgical intervention. Feasibility was confirmed based on the following items: 1. No retrograde infection of the lumbar cistern drainage tube. 2. Cerebrospinal fluid cultures were consistently negative.

### 3.3 Prevention and control of skin lesion infection

Patients with TEN-type drug eruptions are critically ill, with the rapid onset of extensive epidermal detachment and exfoliation. A larger affected skin area is associated with a higher mortality rate (9). Damage to the skin barrier is a primary source of sepsis and bloodstream infections, directly affecting the length of hospital stay and clinical outcomes (10). The patient in this report exhibited epidermal detachment on more than 70% of the body surface. The management of skin lesions was directly linked to the patient’s prognosis. The key nursing measures were as follows (Table 4).

**TABLE 4:** Key points in the care of skin lesion infection.

Nursing measures	Specific content
Laminar flow ward combined with suspension bed	1. Method: the patient was kept in a single-person laminar flow ward with a fluid suspension bed, protective isolation was implemented, and unnecessary visits were restricted. 2. Effect: the laminar flow ward maintained a clean environment, reducing the risk of exogenous infections, and the suspension bed provided an optimal temperature to promote skin recovery (24).
Skin lesion care	1. The patient exhibited more than 70% epidermal detachment across the body. A dorsalis pedis artery puncture catheter and a temperature-monitoring urinary catheter were placed to allow continuous invasive arterial blood pressure and temperature monitoring. This approach decreased the risk of secondary skin damage caused by blood pressure cuffs and mercury thermometers. 2. In addition to routine care, recombinant human acidic fibroblast growth factor was applied four times daily to promote wound healing.
Oral care	1. Cotton swabs soaked in Kangfuxin solution were used for oral care, followed by spraying recombinant human acidic fibroblast growth factor to promote mucosal growth. 2. Once the oral mucosa formed and stabilized, a suction-connected soft-bristle toothbrush was used with chlorhexidine mouthwash to clean the teeth, ensuring oral hygiene and preventing bacterial growth (25).
Perianal care	A stool collection device was used to prevent perianal dermatitis.
Assessment of surgical feasibility	On the 24^th^ day after admission, the patient was transferred to the neurosurgery department for surgical treatment. The outcomes of nursing care were as follows: 1. After 19 days of treatment, most lesions on skin proximal to the knees were dried, affecting 69% of total body surface area (TBSA) as measured by Lund-Browder chart. Re-epithelialization islands are observed in 65% of TBSA, with partial overlap in affected zones, with no secondary infections and complete healing. 2. There was no sign of sepsis. 3. The oral mucosa remained intact and uninfected, with no perianal dermatitis.

### 3.4 Nutritional management

The combined effects of fever, infection, exfoliative dermatitis, and other factors make effective nutritional support critically important for the patient. Concurrently, severe hypoproteinemia may exacerbate cerebral edema, resulting in elevated ICP. For critically ill comatose patients who are unable to eat orally, enteral nutrition support should be prioritized once gastrointestinal function allows, ideally initiated within 24–48 hours of onset (11). Studies have shown (12) that appropriate nutritional support can decrease the risk of postoperative complications among patients with intracerebral hemorrhage and promote immune tolerance and recovery. Moreover, nutritional support plays a vital role in the healing of skin lesions (13). On admission, the patient underwent a nutritional risk screening, which suggested a high risk (NRS2002 score of 5). A nasointestinal tube was placed, and post-pyloric nutritional support was implemented to maintain gastrointestinal function and lower the risk of abdominal distension, nutrient retention, reflux, aspiration, and aspiration pneumonia. Studies have also indicated (8) that the body primarily absorbs proteins in the form of short peptides. Short peptides are short-chain peptides with a size between amino acids and whole proteins. They possess high caloric value, rapid absorption, complete utilization, and no consumption of adenosine triphosphate (ATP). Therefore, the clinical nutrition team recommended enteral nutrition emulsion (short peptide type) at 500 mL per bag, three bags per day (1,500 mL total). The initial feeding rate was 20 mL/h and gradually increased to 60 mL/h if tolerated, ensuring adequate nutrition intake. The patient’s skin lesions healed without reflux, aspiration, or aspiration pneumonia. The NRS2002 score improved to 3, and the patient with his nasointestinal tube was transferred to the neurosurgery department for surgical intervention.

## 4 Discussion

In managing this patient with ICH complicated by TEN, the primary nurse employed advanced health assessment techniques to identify a high risk of infection as the principal nursing diagnosis. This determination was based on the convergence of three pathophysiological vulnerabilities: compromised integumentary barriers due to TEN-induced epidermal detachment, impaired immunological defenses associated with systemic inflammatory response syndrome, and the presence of invasive therapeutic devices, including a tracheostomy and lumbar drain. To mitigate the risk of systemic sepsis, a targeted nursing protocol was implemented across four domains. In respiratory management, high-flow oxygen therapy was used to maintain optimal inspired gas temperature and humidity. A timed, stepwise, segmental suctioning technique was applied to minimize contamination of the tracheostomy tube, reduce cross-infection, and prevent pathogen entry into the lungs. Given the patient’s prolonged bed rest and limited repositioning ability, a bedside nasoenteral tube was inserted for post-pyloric feeding to prevent gastroesophageal reflux and aspiration pneumonia. Antibiotic therapy was escalated from polymyxin B and fluconazole to meropenem and linezolid based on antimicrobial susceptibility testing. Tracheostomy site lesions were managed with phase-specific dressings tailored to denudation, exudation, or crusting phases to prevent exogenous infection. For intracranial infection control, meticulous care of lumbar cisternal drainage was maintained to prevent retrograde infection. In collaboration with neurosurgery and neurology teams, drainage flow was strictly regulated at 200 to 300 mL per day with a uniform velocity, under continuous monitoring of pupils, consciousness, and vital signs. Strict aseptic technique was enforced, and any abnormal drainage prompted immediate specimen collection and clinician notification for dynamic reassessment. In terms of skin management, site- and stage-specific care protocols were applied to all lesions, including those in the oral and perineal regions. To minimize skin trauma while facilitating essential monitoring, a dorsalis pedis arterial catheter and a thermistor-tipped urinary catheter were secured using zero-tension fixation, enabling continuous invasive arterial pressure and core temperature monitoring. A fecal management system was used to prevent perianal dermatitis. Nutritional support was initiated early and progressively increased under the guidance of a dietitian. Administration of 500 mL of short-peptide-based enteral emulsion, given as 1,500 mL per day in three divided doses, provided high-calorie, ATP-sparing nutrition with rapid absorption. Serial NRS-2002 scores improved from 5 to 3. Through synchronized multidisciplinary collaboration and proactive nursing intervention, the patient underwent ventriculoperitoneal shunting on day 24 and was transferred to a rehabilitation facility on day 45 post-admission. This integrated approach enhanced therapeutic efficacy, accelerated recovery, and optimized prognosis.

## 5 Conclusion

The nursing care of patients with intracerebral hemorrhage complicated with severe TEN-type drug eruptions is highly challenging and demands a high level of precision. Scientific and systematic management plays a major role in the recovery of such patients. To address this, future initiatives should advance toward intelligent nursing systems, such as the development of AI-powered early warning models capable of detecting incipient signs of infection through automated skin imaging analysis, thereby enabling rapid, evidence-based interventions (14). Studies have shown (15) that up to 65% of TEN survivors suffer from the symptoms of post-traumatic stress disorder (PTSD), including depression and anxiety. Psychological care was not emphasized during the patient’s stay in our department, and family members were only allowed to encourage the patient through video calls. Psychiatry and psychology specialists should evaluate and address the psychological state of such patients and administer medications when necessary. We recommend formal psychiatric evaluation using ICU-specific psychometric scales, supplemented by virtual reality (VR) therapy to alleviate isolation-induced distress, with pharmacotherapy initiated when indicated. Furthermore, consolidating neurocritical and burn care expertise could establish a goal-directed, stepwise care pathway: neuroprotection → systemic inflammation control → wound regeneration → neurological rehabilitation. This integrated approach warrants further investigation as a potential strategy to expand therapeutic windows and improve survival prospects in such critically complex cases.

## Data Availability

The original contributions presented in this study are included in this article/supplementary material, further inquiries can be directed to the corresponding author.
